# A Shared Regulatory Element Controls the Initiation of *Tcf7* Expression During Early T Cell and Innate Lymphoid Cell Developments

**DOI:** 10.3389/fimmu.2020.00470

**Published:** 2020-03-20

**Authors:** Christelle Harly, Devin Kenney, Yueqiang Wang, Yi Ding, Yongge Zhao, Parirokh Awasthi, Avinash Bhandoola

**Affiliations:** ^1^T-Cell Biology and Development Unit, Laboratory of Genome Integrity, Center for Cancer Research, National Cancer Institute, National Institute of Health, Bethesda, MD, United States; ^2^Université de Nantes, CNRS, Inserm, CRCINA, Nantes, France; ^3^LabEx IGO “Immunotherapy, Graft, Oncology”, Nantes, France; ^4^Typhoon Biotech, BGI-Shenzhen, Shenzhen, China; ^5^Laboratory Animal Sciences Program, Leidos Biomedical Research Inc., Frederick National Laboratory for Cancer Research, National Institute of Health, Frederick, MD, United States

**Keywords:** *Tcf7*, TCF-1, enhancer, T cells, innate lymphoid cells, development

## Abstract

The transcription factor TCF-1 (encoded by *Tcf7*) plays critical roles in several lineages of hematopoietic cells. In this study, we examined the molecular basis for *Tcf7* regulation in T cells, innate lymphoid cells, and migratory conventional dendritic cells that we find express *Tcf7*. We identified a 1 kb regulatory element crucial for the initiation of *Tcf7* expression in T cells and innate lymphoid cells, but dispensable for *Tcf7* expression in *Tcf7*-expressing dendritic cells. Within this region, we identified a Notch binding site important for the initiation of *Tcf7* expression in T cells but not in innate lymphoid cells. Our work establishes that the same regulatory element is used by distinct transcriptional controllers to initiate *Tcf7* expression in T cells and ILCs.

## Introduction

Regulation of gene expression is central to development and cellular differentiation, and erroneous gene expression is linked to many diseases such as cancer. During development, the coordinated and combinatorial action of transcription factors and enhancers results in gene expression patterns that can be distinct between cell lineages and developmental stages. Understanding gene regulation through the study of enhancers is a strategy relevant to understand development and target diseases.

TCF-1 (encoded by the gene *Tcf7*) is an HMG-box transcription factor that plays important functions in development and homeostasis. In particular, TCF-1 is expressed and required at early steps of development of T cells and innate lymphoid cells (ILC) (1–9). The mechanism by which TCF-1 expression is regulated is not understood. Several regulators have been proposed to act upstream of *Tcf7* gene expression, however, whether and how they directly regulate *Tcf7* is unknown.

In this study, we aimed to better understand *Tcf7* gene regulation in hematopoietic lineages. We analyzed publicly available ATAC-seq and ChIP-seq datasets, together with genomic deletions using CRISPR/Cas9 technology, to identify regulatory regions controlling *Tcf7* expression in early stages of T cells and ILC development. We identified a 1kb regulatory element upstream of *Tcf7* that controls the initiation of *Tcf7* expression in T cells and ILCs, but it is dispensable for *Tcf7* expression in conventional Dendritic Cells (cDCs). Within this region, we identified a Notch binding site that contributes to *Tcf7* initiation in T cells but not in ILCs. Our results establish that the many transcriptional similarities between T cells and ILCs include control of *Tcf7* through a shared regulatory element, and further establish that lymphocytes and cDCs differ in the regulatory elements they use to control expression of *Tcf7*.

## Materials and Methods

### Mice

B6-Ly5.2 (CD45.1) mice were from the Jackson Laboratory. *Tcf7*^−^ (7), *Tcf7*^*YFP*^ (2), *Lat*^−^ (10), *Vav1-iCre* (11), *Gata3*^*flox*^ (12) mouse strains have previously been described. Microinjections for *Tcf7*^Δ1−2/Δ1−2^, *Tcf7*^Δ1/Δ1^, *Tcf7*^Δ2/Δ2^, *Tcf7*^Δ3/Δ3^, and *Tcf7*^Δ4/Δ4^ mice were performed on B6 zygotes by co-injecting CRISPR/Cas9 sgRNAs, which flanked either end of each of the deleted regions. *Tcf7*^Δ1−2/Δ1−2^, *Tcf7*^Δ1/Δ1^, and *Tcf7*^Δ2/Δ2^ mice were generated by co-injecting three sgRNAs (guide-1: chr11:52299410-52299431, guide-2: chr11:52306970-52306989, guide-3: chr11:52318934-52318955 on mm10). Cuts made by Guide-1 and Guide-3 resulted in deletion of the entire 20 kb region in *Tcf7*^Δ1−2^ mouse strains; cuts made by Guide-1 and Guide-2 resulted in deletion of the first 8 kb in *Tcf7*^Δ1^ mouse strains; and cuts made by Guide-2 and Guide-3 resulted in deletion of the last 12 kb in *Tcf7*^Δ2^ mouse strains. *Tcf7*^Δ3^ mice were generated by co-injection of two sgRNAs, Guide-3a (chr11: 52313783-52313802) and Guide-3b (chr11: 52314813-52314831). *Tcf7*^Δ4^ mice were generated by co-injection of two sgRNAs, Guide-4a (chr11: 52318337-52318356) and Guide-4b (chr11: 52318905-52318923). *Tcf7*^*NBS*^ mice were generated by microinjection of a single sgRNA (Guide-NBS, chr11: 52314396-52314414) and a 57bp oligonucleotide patch (GAGCATTCTCAGCAGCAGACCCGAGACGTAGTAGCGGCCGCACACGCCACCTTCATA), containing a NotI restriction enzyme site in place of the original NOTCH motif. All CRISPR/cas9 mice were backcrossed to C57BL/6 mice for two (for *Tcf7*^Δ1−2/Δ1−2^, *Tcf7*^Δ1/Δ1^, *Tcf7*^Δ2/Δ2^, *Tcf7*^Δ3/Δ3^, and *Tcf7*^Δ4/Δ4^ mice) or four generations (for *Tcf7*^*NBS*/*NBS*^ mice). To control for off target effects, we compared littermate controls for all new mouse lines to age-matched WT C57BL/6 mice. We found that thymus size, TCF-1 expression in thymocytes, and TCF-1 expression in ILC precursors were similar for all littermate controls and WT C57BL/6 mice. For each deletion, thymus size, TCF-1 expression in thymocytes, and TCF-1 expression in ILC precursors were assessed on 2 or 3 mouse lines generated from independent founders. Figures show the results obtained using one representative mouse line for each deletion. Deletions for these mouse lines were precisely characterized by sequencing the genomic DNA surrounding the enhancer region of interest. The sequences were the following, *Tcf7*^Δ1−2^: …ATGTGGGATTGCTCACGAGACTCTGGCCAAGCACTTAGTG…. (19525bp deletion), *Tcf7*^Δ2^: …GGTAGGTAGGTGCCACCCCTACCTTTTTTAGTAAAAAGCGGACTCTGGCCAAGCAC…. (11971 deletion and 15 bp insertion), *Tcf7*^Δ3^: …TGCTGGGATTAAAGGAATGAGGGCTTGTATAACCTCAGTG…. (1054 bp deletion), *Tcf7*^*NBS*^: see **Figure 7B**. Mice used were 6–10 weeks old and of either sex. Animal procedures were approved by relevant NIH Animal Care and Use Committees. Long-term hematopoietic competitive chimeras were generated by reconstituting CD45.1^+^ recipient mice irradiated at 850 rads, with a mixture of CD45.2^+^ donor cells and CD45.1^+^ competitor cells, either Lin^BM−^ bone marrow (BM) cells or Lin^BM−^Sca-1^+^Kit^hi^ (LSK) cells. Chimeras were analyzed after 12 weeks of reconstitution.

### Antibodies and Flow Cytometry

Cell suspensions were incubated with a mix of rat and mouse serum and hamster IgG, before addition of specific antibodies. Antibodies specific for Ly-6D (49H4), B220 (RA3-6B3), CD19. (1D3), Mac-1 (M1/70), Gr-1 (8C5), CD11c (N418), Ter119 (TER119), NK1.1 (PK136), CD3ε. (2C11), CD8α (53-6.72), CD8β (H35-17.2), CD4 (GK1.5), TCRβ (H57), TCRγδ (GL-3), MHC-II (M5/114.15.2), Kit. (2B8), Sca-1 (D7), Thy-1.2 (53-2.1), α4β7 (DATK32), IL-7Rα (A7R34), 2B4 (eBio244F4), CD25 (PC61.5), CD28 (37.51), CD45.1 (A20), CD45.2 (104), and TOX (TXRX10) were from eBioscience, anti-Flt3 (A2F10) was from BD, anti-TCF-1 (C63D9) was from Cell Signaling. The BM lineage “cocktail” (Lin^BM^) is a mix of the following antibodies: anti-Ly-6D, B220, CD19, Mac-1, Gr-1, CD11c, Ter119, NK1.1, CD3ε, CD8α, CD8β, CD4, TCRβ, and TCRγδ. The T lineage “cocktail” (Lin^T^) is a mix of the following antibodies: anti-B220, CD19, Mac-1, Gr-1, CD11c, Ter119, NK1.1, CD3ε, CD8α, CD8β, TCRβ, and TCRγδ. TCRβ (where specified ic), TOX, and TCF-1 expression were detected by intracellular staining using eBioscience's transcription factor staining buffer set according to the manufacturer's instructions. Live/dead discrimination was performed by staining with DAPI or LIVE/DEAD Fixable Blue (Invitrogen). Samples were acquired using an LSRFortessa flow cytometer (BD) and analyzed using FlowJo software (Tree Star). All analyses are presented on singlet live cells. BM progenitors were sorted using an Aria flow cytometer (BD). Absolute cell counts were obtained using an Accuri C6 plus (BD). Thymocyte populations are defined as followed: Early T cell precursor (ETP) (Lin^T−^Kit^hi^CD25^−^, see **Figure 2A**), Double Negative (DN)2 (Lin^T−^Kit^hi^CD25^+^, see **Figure 2A**), DN3a (Lin^T−^Kit^lo^CD25^+^CD28^lo^), DN3b (Lin^T−^Kit^low^CD25^+^CD28^hi^), Immature Single Positive (ISP) (CD8α^+^CD4^−^CD3ε^−^), Double Positive (DP) (CD8α^+^CD4^+^), mature CD4 (CD8α^−^CD4^+^TCRβ^+^), mature CD8 (CD8α^+^CD4^−^TCRβ^+^). BM populations are defined as followed: Lymphoid-primed Multipotent Progenitor (LMPP) (Lin^BM−^Kit^hi^Sca-1^+^Flt3^hi^), LSK (Lin^BM−^Sca1^+^Kit^hi^), TCF-1^+^ Early Innate Lymphoid Progenitor (EILP) (Lin^BM−^Kit^+^α4β7^+^2B4^+^Thy1.2^−^TCF-1^+^, see **Figure 3A**), ILC Precursor (ILCP) (Lin^BM−^Kit^+^α4β7^+^2B4^+^Flt3^−^Thy1.2^+^IL-7Rα^+^, see Supplementary Figure 2A), ILC2 progenitor (ILC2P) (Lin^BM−^Kit^−^2B4^lo^Thy1.2^hi^IL-7Rα^+^, see Supplementary Figure 2B) TOX^+^ EILP (Lin^BM−^Kit^+^α4β7^+^2B4^+^Thy1.2^−^TOX^+^, see **Figure 3E**).

### Bioinformatic Analysis

**Figure 1:**
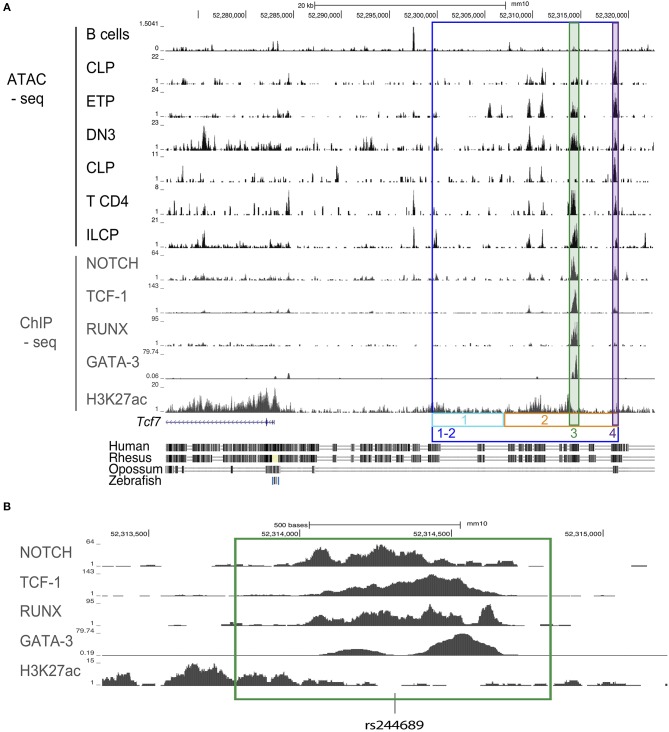
Design schematic for CRISPR/cas9 guides for mice generation. **(A)** ATAC-seq profiles of the region upstream of the *Tcf7* locus in B cells, CLP, ETP, DN3, CD4 T cells, and ILCP; ChIP-seq profiles for Notch in a T cell line, TCF-1 in thymocytes, RUNX in thymocytes, GATA-3 in DP, and H3K27ac in naïve CD4 T cells. Colored, boxed regions represent regions targeted for deletion with CRISPR/cas9 guides. Conservation tracks are displayed. **(B)** Focused view of transcription factor binding within the deleted region 3 (green). Location of a common single nucleotide polymorphism (SNP) [rs244689] within this region is marked.

Common Lymphoid progenitor (CLP), CD4, ILCP (GSE98662) (13) ATAC-seq data and TCF-1 (GSE46662) (14, 15), RUNX (GSE33653) (16), GATA-3 (GSE31235) (17), Notch1 (GSE61504) (18), and H3K27ac (GSE76031) (19) ChIP-seq data were downloaded from the NCBI SRA database and aligned to mm10 mouse genome using bowtie2 (Version 2.3.4.2). B cells, CLP, ETP, DN3, and cDCs ATAC-seq data were downloaded from ImmGen (GSE100738) (20). Datasets were viewed and analyzed using the UCSC Genome Browser (http://genome.ucsc.edu/) (21) (Figure 1) or IGV (22) (Supplementary Figure 1). Motif analysis was done using the ECR Browser (23).

### *In vitro* Culture Experiments

LMPP were cultured on irradiated OP9 stromal layers expressing the Notch ligand DL1 (24) in α-MEM media (Gibco) supplemented with 20% FBS (Atlanta Biologicals), glutamine (Gibco), penicillin and streptomycin (Gibco), Flt3-L. (10 ng/mL, PeproTech) and IL-7 (5 ng/mL, PeproTech). CD45.2^+^GFP^−^ cells were considered for analysis of hematopoietic cells.

### Statistical Analysis

Statistical analysis was performed on groups with limited variance using Excel or Prism. Differences between groups of mice or wells were determined by a two-tailed unpaired Student's *t*-test. A *p* of *p* < 0.05 was considered statistically significant. Sample sizes were empirically determined, no samples or animals were excluded from the analysis, no randomization, or blinding was used.

## Results

### Identification of Candidate Regulatory Regions Upstream of *Tcf7*

We wished to identify candidate regulatory regions that might be required for *Tcf7* expression in one or several hematopoietic lineages. Assay for transposase accessible chromatin followed by next generation sequencing (ATAC-seq) reveals regions of open chromatin, and thus can be used to identify putative enhancers. Using publicly available ATAC-seq data, we investigated the presence of open chromatin regions in T cell precursors (ETP, DN3), mature T cells (CD4), and ILC precursors (ILCP), which all express *Tcf7*; and B cells, that lack *Tcf7*. We additionally examined common lymphoid progenitors (CLP), which lack *Tcf7* expression, and can give rise to T cells, B cells, and ILCs. This analysis identified a 20 kb region upstream of the *Tcf7* promoter that showed peaks of open chromatin shared by all *Tcf7*-expressing populations and in a lesser extent CLP, but not B cells (Figure 1A). On the other hand, the *Tcf7* super-enhancer previously identified in T cells (25) and located downstream of the region 1-2, presented numerous ATAC-seq peaks that were not shared between *Tcf7*-expressing populations, and were not specifically present in *Tcf7*-expressing populations compared to B cells (Supplementary Figure 1). Importantly, most of the ATAC-seq peaks present in region 1-2 were located in highly conserved regions between mouse and human and contained many binding motifs for factors involved in hematopoiesis (Figure 1A, and not shown). Interestingly, one of the conserved ATAC-seq peaks within the 20 kb region contained a single nucleotide polymorphism (rs244689), that is linked to systemic lupus erythematosus (SLE) in Asian populations (Figure 1B) (26).

We further examined whether candidate controllers of *Tcf7* bind in the vicinity of the *Tcf7* locus. Although *Tcf7* controllers are yet to be identified in ILC, many transcription factors have been proposed to act upstream of *Tcf7* expression in developing T cells. *Tcf7* expression is thought to initiate downstream of Notch1 signaling in T cell precursors (1, 8, 27), and this initiation requires RUNX factors (28). GATA-3 and TCF-1 itself might further contribute to optimal expression of *Tcf7* (8, 29). We used publicly available ChIP-seq data for these transcriptional controllers in T-lineage cells, and found that they all bind the 20 kb candidate regulatory region we identified (Figures 1A,B). On the other hand, we did not observe binding for these factors in the *Tcf7* super-enhancer (Supplementary Figure 1). Importantly, ChIP-seq peaks for Notch1, TCF-1, RUNX, and GATA-3 (Figure 1B) all co-localized with binding motifs for these factors that were conserved between mouse and human (not shown). We therefore hypothesized that the 20 kb 1-2 region contains regulatory elements important for *Tcf7* expression in one or several hematopoietic lineages.

### The Region 1-2 Is Required for *Tcf7* Expression in the T Cell Lineage

**Figure 2:**
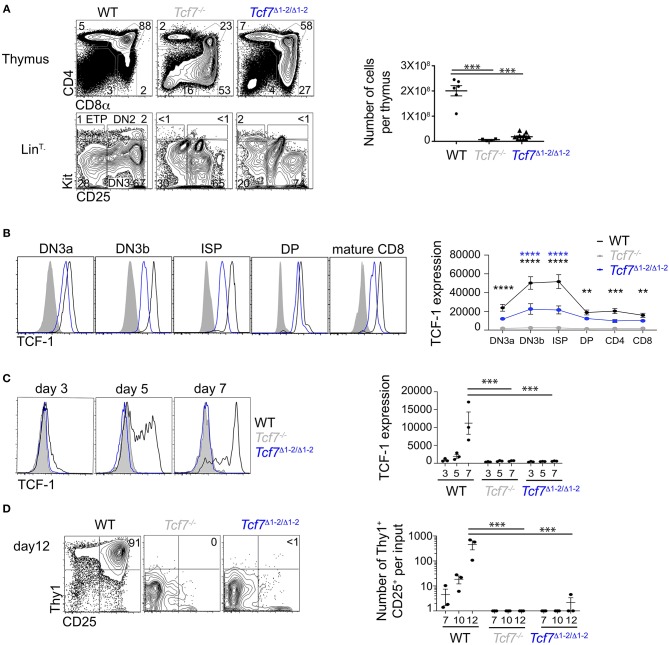
Defect in TCF-1 expression during early T cell development in *Tcf7*^Δ1−2/Δ1−2^ mice. **(A,B)** Flow cytometric analysis of *Tcf7*^Δ1−2/Δ1−2^, *Tcf7*^−/−^, and WT thymocytes. **(A)** Representative flow plots are shown for total thymocytes (top, left) and Lin^T−^ thymocytes (bottom, left). Quantification of total thymocytes is shown for 6 WT mice, 4 *Tcf7*^−/−^ mice, and 10 *Tcf7*^Δ1−2/Δ1−2^ mice pooled from three independent experiments (right). Data are presented as average +/– SEM. A two-tailed Student's *t*-test was used to determine significance. ***p < 0.005. **(B)** TCF-1 intracellular staining on thymocytes subsets. Representative flow plots are shown (left). Data are presented as average of TCF-1 geometric mean fluorescence intensity (gmfi) +/– SEM for *n* = 4 mice analyzed in one experiment (right). Data are representative of three independent experiments. A two-tailed Student's *t*-test was used to determine significance. **p < 0.01, ***p < 0.001, ****p < 0.0001 **(C,D)** Flow cytometric analysis of Lymphoid-primed multipotent progenitors (LMPP) isolated from *Tcf7*^Δ1−2/Δ1−2^, *Tcf7*^−/−^, and WT mice and cultured in the presence of Flt3 and IL7. **(C)** TCF-1 expression on day 3, 5, and 7 of culture. Representative flow plots are shown (left). Data are presented as average of TCF-1 gmfi +/- SEM for *n* = 3 well in one experiment (right). A two-tailed Student's *t*-test was used to determine significance. ***p < 0.001. Data are representative of three independent experiments. **(D)** Flow cytometric profile on day12 of culture (left). Quantification of T-lineage cells (Thy1^+^*CD*25^+^) number per input on day 7, 10, and 12 of culture. Data are presented as average +/- SEM for *n* = 3 well in one experiment. A two-tailed Student's *t*-test was used to determine significance. ***p < 0.001. Data are representative of three independent experiments.

Using CRISPR/Cas9, we generated mice lacking the 20 kb region 1-2, called the *Tcf7*^Δ1−2^ strain. To examine whether the deletion of the region 1-2 affected *Tcf7* expression in thymocytes, we first examined T cell development in the *Tcf7*^Δ1−2/Δ1−2^ mice compared to *Tcf7*^−/−^ mice that lack TCF-1 functional protein (7). Similarly to *Tcf7*^−/−^ mice, *Tcf7*^Δ1−2/Δ1−2^ mice presented a reduced frequency of CD4^+^CD8α^+^ DP cells, and total thymocyte numbers were decreased more than 10-fold compared to WT mice (Figure 2A). Early T cell development was particularly disrupted compared to WT cells, as shown by the absence of recognizable Lin^T−^Kit^hi^*CD*25^−^ ETP and Lin^T−^Kit^hi^CD25^hi^ DN2 subsets and development of aberrant Lin^T−^Kit^int^CD25^int^ subsets (Figure 2A).

We next analyzed levels of TCF-1 protein by intracellular staining on thymocytes. TCF-1 was detectably expressed in *Tcf7*^Δ1−2/Δ1−2^ thymocytes from DN3 onward, although at much lower levels compared to WT (Figure 2B). Dynamic changes in TCF-1 expression were greatly attenuated in *Tcf7*^Δ1−2/Δ1−2^, most notably after β-selection when TCF-1 expression normally peaks during T cell development (Figure 2B). Interestingly, TCF-1 expression never reached WT levels, even in peripheral T cell populations (Figure 2B, not shown).

We further wished to examine *Tcf7* initiation during T cell development. Dramatic defects in the generation of ETP and DN2 cells in *Tcf7*^Δ1−2/Δ1−2^ mice prevented direct assessment of TCF-1 expression at these early developmental stages. We therefore examined TCF-1 initiation in *Tcf7*^Δ1−2/Δ1−2^ T cell precursors *in vitro*, after culture on stromal layers expressing Notch ligand (OP9-DL1). In this system, WT BM progenitors upregulated TCF-1 by day 5 (Figure 2C) and generated Thy1^hi^CD25^hi^ DN2/DN3 stages T-lineage cells by day 7 (Figure 2D). On the other hand, *Tcf7*^Δ1−2/Δ1−2^ and *Tcf7*^−/−^ progenitors failed to upregulate TCF-1 and to generate DN2/DN3 T-lineage cells, even at later time points in the culture (Figures 2C,D). Altogether, these results indicate that the 20 kb region deleted in *Tcf7*^Δ1−2/Δ1−2^ mice is required for initiation of *Tcf7* expression at early stages of T cell development.

### The Region 1-2 Is Required for *Tcf7* Expression in the ILC Lineage

**Figure 3:**
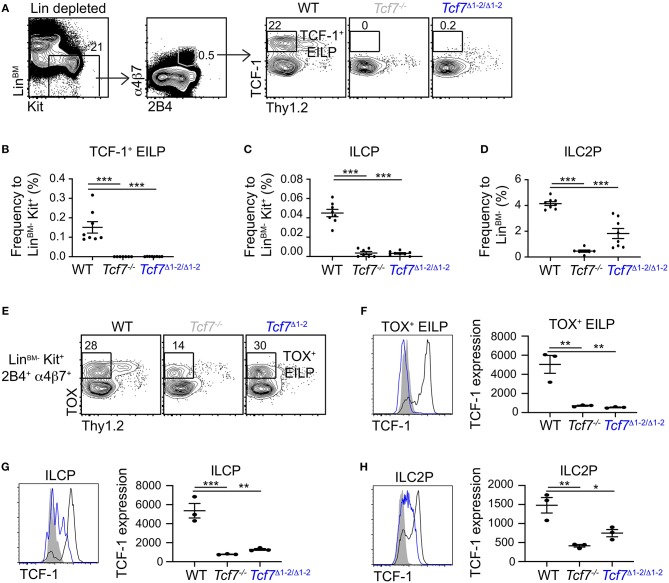
Defect in TCF-1 expression during early ILC development in *Tcf7*^Δ1−2/Δ1−2^ mice. Flow cytometric analysis of *Tcf7*^Δ1−2/Δ1−2^, *Tcf7*^−/−^, and WT BM cells. **(A)** Representative gating strategy used to identify TCF-1^+^ EILP (Lin^BM−^Kit^+^α4β7^+^2B4^+^Thy1.2^−^TCF-1^+^). **(B–D)** Quantification of frequencies of **(B)** TCF-1^+^ EILP, **(C)** ILCP (Lin^BM−^Kit^+^α4β7^+^2B4^+^Flt3^−^Thy1.2^+^IL-7Rα^+^, see Supplementary Figure 2A) and **(D)** ILC2P (Lin^BM−^Kit^−^2B4^lo^Thy1.2^hi^IL-7Rα^+^, see Supplementary Figure 2B) is shown for 8 WT mice, 7 *Tcf7*^−/−^ mice, and 8 *Tcf7*^Δ1−2/Δ1−2^ mice pooled from three independent experiments. Data are presented as average +/– SEM. A two-tailed Student's *t*-test was used to determine significance. ***p < 0.005. **(E)** Representative gating strategy used to define TOX^+^ EILP (Lin^BM−^Kit^+^α4β7^+^2B4^+^Thy1.2^−^TOX^+^), showing Lin^BM−^Kit^+^α4β7^+^2B4^+^ cells. **(F–H)** TCF-1 protein expression in **(F)** TOX^+^ EILP, **(G)** ILCP and **(H)** ILC2P. Representative flow plots are shown (left). Data are presented as average of TCF-1 gmfi +/– SEM for *n* = 3 mice analyzed in one experiment (right). A two-tailed Student's *t*-test was used to determine significance. *p < 0.05, **p < 0.01, ***p < 0.005. Data are representative of three independent experiments.

We next asked whether the 20 kb region 1-2 was also important for *Tcf7* expression in the ILC lineage. We examined the presence of TCF-1-expressing ILC precursors in the BM of *Tcf7*^Δ1−2/Δ1−2^ mice (30). The frequency of TCF-1-expressing early innate lymphoid progenitors (EILP) that initiate *Tcf7* expression (9) was dramatically decreased in *Tcf7*^Δ1−2/Δ1−2^ mice compared to WT mice (Figures 3A,B). Frequencies of the later ILC precursors, ILCP (31) and ILC2P (32) were also decreased in *Tcf7*^Δ1−2/Δ1−2^ mice compared to WT mice, consistent with a defect in *Tcf7* expression in the ILC lineage (Figures 3C,D) (2, 3, 6, 9).

To investigate whether ILC precursors still develop in *Tcf7*^Δ1−2/Δ1−2^ mice but fail to express TCF-1, we visualized EILP using TOX intracellular staining instead of TCF-1 (2). This alternative gating strategy revealed that, similarly to *Tcf7*^−/−^ mice, some EILP were still present in *Tcf7*^Δ1−2/Δ1−2^ mice (Figure 3E), but they lacked TCF-1 expression (Figure 3F). TCF-1 expression was also greatly reduced on the rare ILCP and ILC2P that developed in *Tcf7*^Δ1−2/Δ1−2^ mice (Figures 3G,H). These results indicate that the 20 kb region deleted in *Tcf7*^Δ1−2/Δ1−2^ mice is required for initiation of *Tcf7* expression at early stages of ILC development.

### The Region 1-2 Is Dispensable for *Tcf7* Expression in Migratory cDC

**Figure 4:**
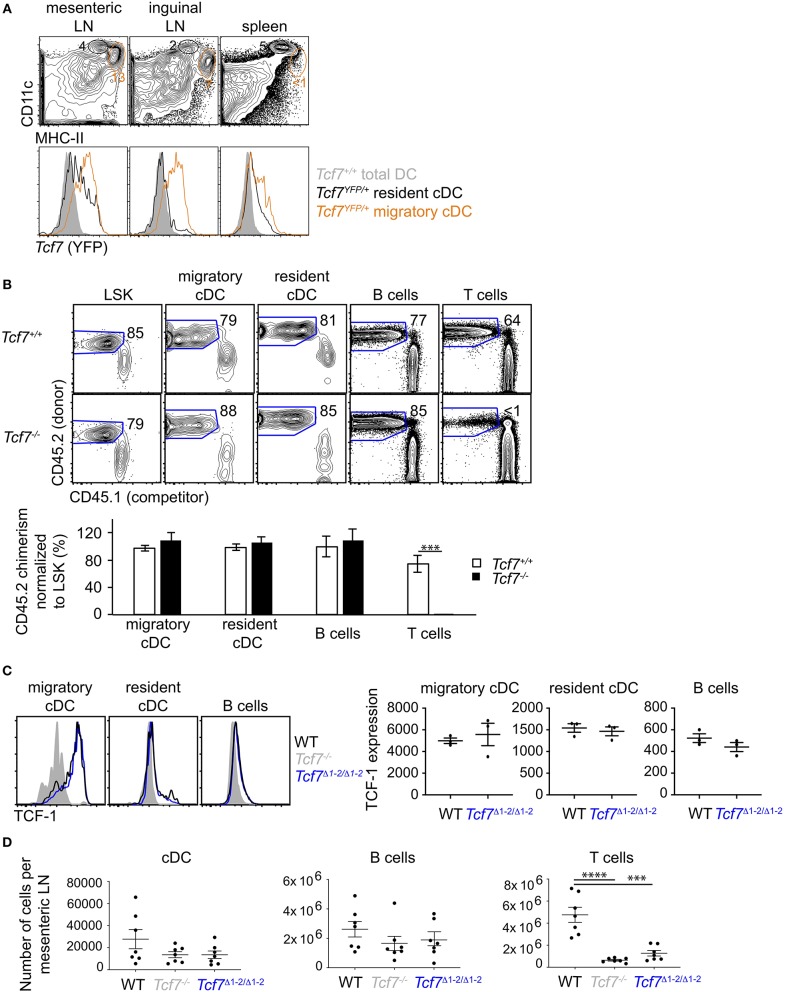
The region 1–2 is not required for TCF-1 expression in migratory cDC. **(A)** Flow cytometric analysis of spleen and LN from *Tcf7*^*YFP*/+^ and WT mice. Plots are gated on TCR^−^CD19^−^ cells and show CD11c^hi^HMCII^lo^ resident cDCs (black) and CD11c^lo^HMCII^hi^ migratory cDCs (orange) (top), and their *Tcf7* (YFP) expression compared to total DC (gray) in *Tcf7*^+/+^ mice. **(B)** Flow cytometric analysis of long-term hematopoietic competitive chimeras reconstituted with Lin^BM−^ BM cells from *Tcf7*^−/−^ or WT CD45.2^+^ littermates mixed with CD45.1^+^ Lin^BM−^ BM cells (3:1 ratio). LSK are from the BM, migratory and resident cDC, B cells and T cells are analyzed in the mesenteric LN. Representative flow plots are shown (top). Data are presented are average +/– SEM for 12 *Tcf7*^+/+^ and 11 *Tcf7*^−/−^ chimeric mice pooled from three independent experiments (bottom). A two-tailed Student's *t*-test was used to determine significance. ***p < 0.005. **(C)** Flow cytometric analysis of TCF-1 protein expression in migratory and resident cDC, and B cells from mesenteric LN of *Tcf7*^Δ1−2/Δ1−2^, *Tcf7*^−/−^, and WT mice. Representative flow plots are shown (left). Data are presented as average of TCF-1 gmfi +/– SEM for *n* = 3 mice analyzed in one experiment (right). A two-tailed Student's *t*-test was used to determine significance. Data are representative of three independent experiments. **(D)** Flow cytometric analysis of mesenteric LN cells quantifying cDCs, B cells, and T cells. Data are presented as average +/– SEM for 7 mice of each genotype pooled from three independent experiments. A two-tailed Student's *t*-test was used to determine significance. ***p < 0.001, ****p < 0.0001.

We additionally examined TCF-1 expression in other hematopoietic lineages. Using a *Tcf7*-reporter mouse that expresses the Yellow Fluorescent Protein (YFP) downstream of the endogenous *Tcf7* gene (2), we found that *Tcf7* expression was evident in cDCs present in lymph nodes (LN) but very low in spleen cDCs. *Tcf7*-expressing cDCs present in LN corresponded to migratory DCs as defined using CD11c and MHC-II expression (33), whereas such cells were almost absent from the spleen (Figure 4A). To examine whether TCF-1 played a role in the development of migratory cDCs, we reconstituted lethally irradiated mice with Lin^BM^-depleted BM cells isolated from *Tcf7*^−/−^ CD45.2^+^ mice or littermate control, mixed with CD45.1^+^ Lin^BM^-depleted BM cells. The competitive setting of this experiment should reveal even mild defects in hematopoietic development. After 12 weeks of reconstitution, migratory and resident cDC developed from *Tcf7*^−/−^ hematopoietic precursors as efficiently as WT cells, similarly to B cells that do not express or require TCF-1 (Figure 4B). In contrast, T cell reconstitution from *Tcf7*^−/−^ hematopoietic precursors was greatly deficient as expected (Figure 4B). We next examined whether the region 1–2 was required for *Tcf7* expression in LN cDCs. TCF-1 expression in LN cDCs as well as cDC numbers appeared unaffected by deletion of the region 1-2 (Figures 4C,D). We did not observe ectopic expression of TCF-1 in *Tcf7*^Δ1−2/Δ1−2^ mice, in lineages normally lacking TCF-1, like B cells (Figure 4C). The region 1-2 appears therefore dispensable for *Tcf7* expression in migratory cDCs, and for *Tcf7* lack of expression in B cells. Consistently, regions of open chromatin were absent from the region 1-2 in cDCs (Supplementary Figure 1).

### A 1kb Region Is Required for *Tcf7* Initiation in Early T Cell and ILC Precursors

To refine the region responsible for *Tcf7* initiation in T cell and ILC lineages, we subdivided the 20 kb 1-2 region into a 8kb (region 1) and a 12 kb (region 2) regions (Figure 1A). We assessed mice deficient for each of these regions (*Tcf7*^Δ1/Δ1^ and *Tcf7*^Δ2/Δ2^ mice, respectively) for defects in T cell and ILC development. No apparent defect in T cell development was seen in *Tcf7*^Δ1/Δ1^ mice (Supplementary Figures 3A,C). Furthermore, although region 1 included a region previously proposed to play key functions for *Tcf7* expression specifically in naïve T cells (19) (Supplementary Figure 1), no defect was seen for TCF-1 expression in *Tcf7*^Δ1/Δ1^ mice (Supplementary Figure 3D). In EILP, *Tcf7*^Δ1/Δ1^ mice did not show a significant defect in TCF-1 initiation (Supplementary Figures 4A,D). On the other hand *Tcf7*^Δ2/Δ2^ mice showed major defects in T cell and ILC development, similar to *Tcf7*^Δ1−2/Δ1−2^ mice (Supplementary Figures 3A,C, 4A,D). These data indicate that a key regulatory element controlling *Tcf7* initiation during early T cell and ILC development falls within region 2, and that region 1 is largely dispensable for *Tcf7* initiation in these two lineages.

**Figure 5:**
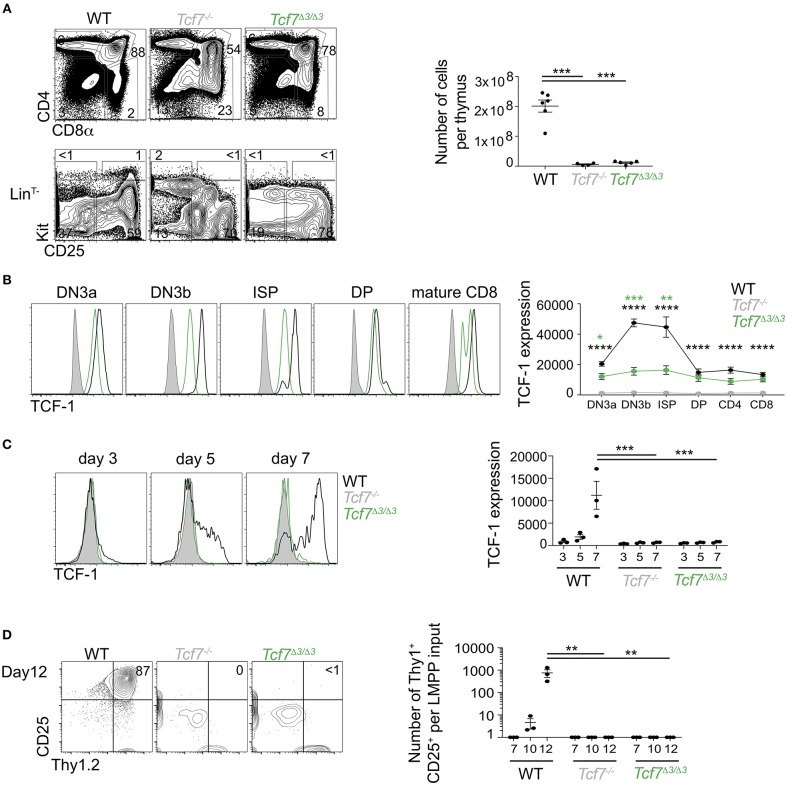
Defect in TCF-1 expression during early T cells development in *Tcf7*^Δ3/Δ3^ mice. **(A,B)** Flow cytometric analysis of *Tcf7*^Δ3/Δ3^, *Tcf7*^−/−^, and WT thymocytes. **(A)** Representative flow plots are shown for total thymocytes (top, left) and Lin^T−^ thymocytes (bottom, left). Quantification of total thymocytes is shown for 6 WT mice, 4 *Tcf7*^−/−^ mice, and 5 *Tcf7*^Δ3/Δ3^ mice pooled from three independent experiments (right). Data are presented as average +/– SEM. A two-tailed Student's *t*-test was used to determine significance. ***p < 0.005. **(B)** TCF-1 intracellular staining on thymocytes subsets. Representative flow plots are shown (left). Data are presented as average of TCF-1 gmfi +/– SEM for *n* = 3 mice analyzed in one experiment (right). Data are representative of three independent experiments. A two-tailed Student's *t*-test was used to determine significance. ***p < 0.001 **(C,D)** Flow cytometric analysis of Lymphoid-primed multipotent progenitors (LMPP) isolated from *Tcf7*^Δ3/Δ3^, *Tcf7*^−/−^, and WT mice and cultured in the presence of Flt3 and IL7. **(C)** TCF-1 expression on day 3, 5, and 7 of culture. Representative flow plots are shown (left). Data are presented as average of TCF-1 gmfi +/– SEM for *n* = 3 well in one experiment (right). A two-tailed Student's *t*-test was used to determine significance. ***p < 0.001 Data are representative of three independent experiments. **(D)** Flow cytometric profile on day12 of culture (left). Quantification of T-lineage cells (Thy1^+^CD25^+^) number per input on day 7, 10, and 12 of culture. Data are presented as average +/– SEM for *n* = 3 well in one experiment. A two-tailed Student's *t*-test was used to determine significance. ***p < 0.001. Data are representative of three independent experiments. *p <0.05; ** *p* <0.01; ****p < 0.0001.

**Figure 6:**
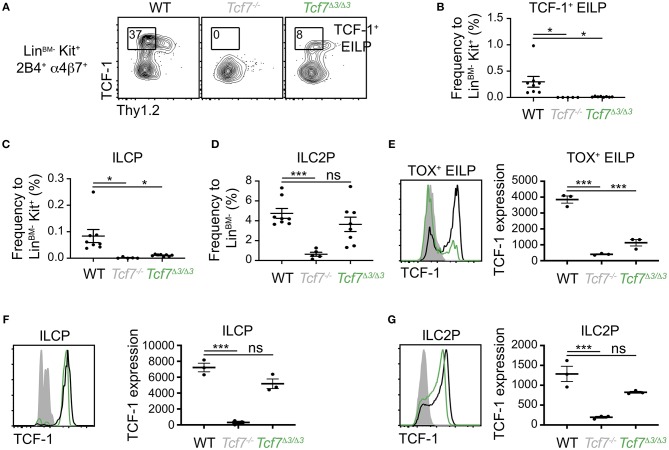
Defect in TCF-1 expression during early ILC development in *Tcf7*^Δ3/Δ3^ mice. Flow cytometric analysis of *Tcf7*^Δ3/Δ3^, *Tcf7*^−/−^, and WT BM cells. **(A)** Representative gating strategy showing TCF-1^+^ EILP, gated on Lin^BM−^*Kit*^+^α4β7^+^2*B*4^+^ cells. **(B–D)** Quantification of frequencies of **(B)** TCF-1^+^ EILP, **(C)** ILCP and **(D)** ILC2P is shown for 8 WT mice, 5 *Tcf7*^−/−^ mice, and 8 *Tcf7*^Δ3/Δ3^ mice pooled from three independent experiments. Data are presented as average +/– SEM. A two-tailed Student's *t*-test was used to determine significance. *p < 0.05, ***p < 0.005. **(E–G)** TCF-1 protein expression in **(E)** TOX^+^ EILP, **(F)** ILCP and **(G)** ILC2P. Representative flow plots are shown (left). Data are presented as average of TCF-1 gmfi +/– SEM for *n* = 3 mice analyzed in one experiment (right). A two-tailed Student's *t*-test was used to determine significance. ***p < 0.005. Data are representative of three independent experiments.

Further CRISPR deletions were performed on regions containing two highly conserved ATAC-seq peaks found within the 12 kb region 2; a 1 kb region (region 3, deleted in the *Tcf7*^Δ3^ mouse strain) and a 600 bp region (region 4, deleted in *Tcf7*^Δ4^ mouse strain) (Figure 1). No apparent phenotype was observed in *Tcf7*^Δ4/Δ4^ mice for T cell development (Supplementary Figures 3B,C). In contrast, *Tcf7*^Δ3/Δ3^ mice showed major defects in thymic populations and cellularity similar to the *Tcf7*^Δ1−2/Δ1−2^ and *Tcf7*^−/−^ mice (Figures 2A, 5A). Intracellular staining for TCF-1 in *Tcf7*^Δ3/Δ3^ mice showed TCF-1 expression pattern similar to that seen in *Tcf7*^Δ1−2/Δ1−2^ mice (Figures 2B, 5B). Short term *in vitro* culture of *Tcf7*^Δ3/Δ3^ hematopoietic precursors on stromal layers expressing Notch ligand (OP9-DL1) showed that TCF-1 expression and early T cell development failed to be initiated, similarly to what was seen with *Tcf7*^Δ1−2/Δ1−2^ hematopoietic precursors (Figures 5C,D). Similarly, in EILP, TCF-1 expression was not significantly affected in *Tcf7*^Δ4/Δ4^ mice (Supplementary Figures 4A,D) whereas it was greatly deficient in *Tcf7*^Δ3/Δ3^ mice (Figures 6A–E).

Interestingly, although the *Tcf7*^Δ1/Δ1^ and *Tcf7*^Δ4/Δ4^ mice did not show significant defects in TCF-1 expression at EILP stage, defects in TCF-1 expression were evident at later stages of ILC development, in ILCP and ILC2P (Supplementary Figures 4E,F). This result indicated that regions 1 and 4 might regulate *Tcf7* expression after initiation. On the other hand, in *Tcf7*^Δ1−2/Δ1−2^, *Tcf7*^Δ2/Δ2^, and *Tcf7*^Δ3/Δ3^ mice that presented dramatic defects in TCF-1 initiation of expression at EILP stage, TCF-1 expression showed upregulation at later developmental stages and almost reached WT levels (Figures 3G,H, 6F,G and Supplementary Figures 4E,F). Consistently, despite the dramatic defects in EILP and ILCP numbers (Figures 3A–C, 4A–C, Supplementary Figures 4A,B), ILC2P still developed in *Tcf7*^Δ1−2/Δ1−2^, *Tcf7*^Δ2/Δ2^, and *Tcf7*^Δ3/Δ3^ mice (Figures 3D, 4D, Supplementary Figure 4C). This upregulation could indicate that region 3 is dispensable for TCF-1 regulation of expression after initiation. Alternatively, TCF-1 upregulation could be the result of compensatory mechanisms that are secondary to the defect in *Tcf7* initiation.

Altogether, the data indicate that the 1 kb region 3 contains regulatory elements critical for the initiation of *Tcf7* expression in the T cell and ILC lineages. On the other hand, regions 1 and 4 are largely dispensable for *Tcf7* initiation of expression in T cells and ILCs, but may play important functions at later developmental stages in ILC.

### Disruption of a Notch Binding Site Upstream of *Tcf7* Impact TCF-1 Expression in T Cells

**Figure 7:**
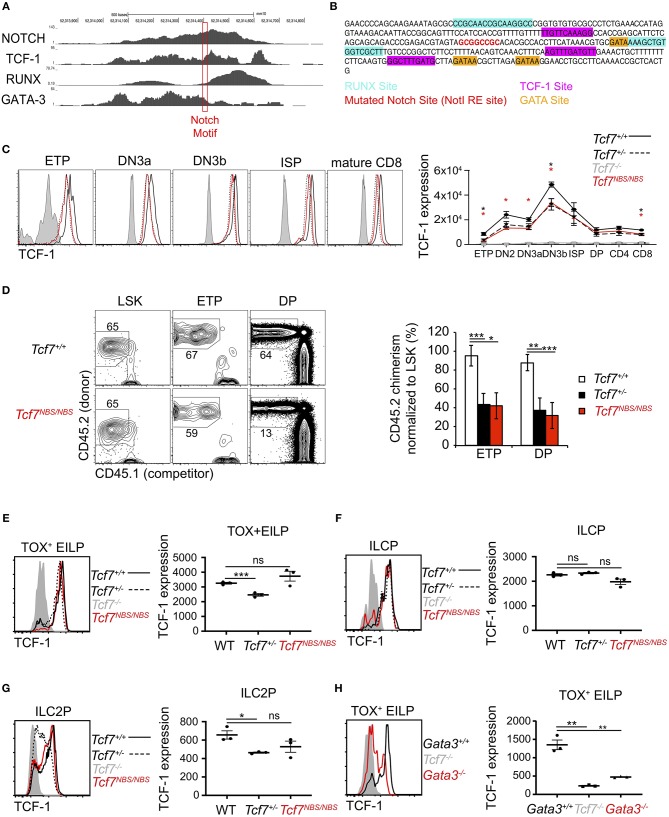
Involvement of a Notch binding site in TCF-1 initiation during early T cell development. **(A)** ChIP-seq profiles for transcription factors binding in region 3. The red box shows the location of a Notch motif targeted in the *Tcf7*^*NBS*^ mouse strain. **(B)** DNA sequencing showing the mutated Notch binding site (red) in the *Tcf7*^*NBS*^ mouse strain. Unaffected surrounding GATA (orange), RUNX (blue) and TCF-1 (pink) binding sites are shown. **(C)** TCF-1 intracellular staining on thymocytes subsets (left) and quantification of TCF-1 protein expression (right). Data are pooled from two independent experiments and presented as average +/– SEM, for 4 WT mice, 4 *Tcf7*^+/−^ mice, 4 *Tcf7*^*NBS*/*NBS*^ mice, and 3 *Tcf7*^−/−^ mice. A two-tailed Student's *t*-test was used to determine significance. *p < 0.05. Data are representative of three independent experiments **(D)** Flow cytometric analysis of thymocytes from long-term hematopoietic competitive chimeras generated by co-injecting LSK from CD45.1^+^ (competitor) and WT, *Tcf7*^+/−^, or *Tcf7*^*NBS*/*NBS*^ CD45.2^+^ (donor) mice into irradiated CD45.1^+^ host at a ratio of 1:2. Representative flow plot (left) and quantification of percent donor cells normalized to LSK (right) are shown. Data are presented as average +/– SEM for 9 WT mice, 7 *Tcf7*^+/−^ mice, and 8 *Tcf7*^*NBS*/*NBS*^ chimeric mice pooled from three independent experiments. A two-tailed Student's *t*-test was used to determine significance. *p < 0.05, **p < 0.01, ***p < 0.005. **(E–G)** TCF-1 protein expression in **(E)** TOX^+^ EILP, **(F)** ILCP, and **(G)** ILC2P. Representative flow plots are shown (left). Data are presented as average of TCF-1 gmfi +/– SEM for *n* = 3 mice analyzed in one experiment (right). A two-tailed Student's *t*-test was used to determine significance. *p < 0.05, ***p < 0.005. Data are representative of three independent experiments. **(H)** Flow cytometric analysis of TCF-1 protein expression in TOX^+^ EILP from *Tcf7*^−/−^, *Vav1-iCre*^+^ *Gata3*^*f*/*f*^ (called *Gata3*^−/−^), and *Vav1-iCre*^+^ *Gata3*^+/+^ (called *Gata3*^+/+^) mice. Representative flow plots are shown (left). Data are presented as average of TCF-1 gmfi +/– SEM for *n* = 3 mice analyzed in one experiment (right). A two-tailed Student's *t*-test was used to determine significance. **p < 0.01. Data are representative of three independent experiments.

The 1 kb region 3 we identified as crucial for *Tcf7* expression during T cell and ILC development was bound by several transcription factors previously proposed to play important functions in regulating *Tcf7* expression in T cells, namely Notch, TCF-1, RUNX, and GATA-3 (Figure 7A). This region further includes binding motifs for these factors that are conserved between mouse and human (Figure 7B). In particular, a Notch/Rbpjk binding motif was previously proposed to control *Tcf7* initiation in T cells (1, 8) (Figure 7A). Using CRISPR/Cas9 technology, we generated a mouse selectively lacking this Notch binding site (NBS, *Tcf7*^*NBS*/*NBS*^) (Figure 7B). Thymocyte populations and cellularity appeared unaffected in these mutant mice (Supplementary Figure 5A). However, TCF-1 expression was decreased by about 2-fold at every stage of T cell development, to levels similar to *Tcf7*^+/−^ heterozygous mice (Figure 7C). We further examined T cell development from CD45.2^+^ *Tcf7*^*NBS*/*NBS*^ hematopoietic progenitors (Lin^BM−^Kit^hi^Sca1^+^; LSK) in the presence of WT CD45.1^+^ competitor cells in long-term hematopoietic chimeras. After 10 weeks of reconstitution, we quantified the percentage of CD45.2^+^ donor cells in thymocyte populations compared to LSK. The percentage of cells generated by *Tcf7*^*NBS*/*NBS*^ hematopoietic progenitors significantly decreased from LSK to ETP compared to WT cells, similarly to *Tcf7*^+/−^ hematopoietic progenitors (Figure 7D). Interestingly, unlike the larger 1 kb region 3, the NBS deletion affected TCF-1 expression at all stages of T cell development evenly, without particularly impacting TCF-1 upregulation at β-selection (Figure 7C and Supplementary Figure 5B). This result suggested that Notch signaling is not responsible for this upregulation, and that other factors are controlling *Tcf7* upregulation at this transition. Because pre-TCR signaling is a major driver of changes occurring at β-selection, pre-TCR signaling could be important in inducing TCF-1 upregulation at the TCRβ-selection checkpoint. Consistent with this hypothesis, DN3 thymocytes expressing intracellular TCRβ did not upregulate TCF-1 in the absence of LAT, a signaling molecule required downstream of the pre-TCR (Supplementary Figure 5C). Finally, we examined whether the NBS played some function in *Tcf7* expression in the ILC lineage. ILC precursors quantification showed no significant differences between *Tcf7*^*NBS*/*NBS*^, *Tcf7*^+/−^, and WT mice (Supplementary Figures 5D–F). We next examined TCF-1 expression in *Tcf7*^*NBS*/*NBS*^ ILC precursors compared to *Tcf7*^+/−^ and WT. Although TCF-1 expression was detectably lower in *Tcf7*^+/−^ EILP and ILC2P compared to WT, TCF-1 expression by *Tcf7*^*NBS*/*NBS*^ ILC precursors was similar to WT (Figures 7E–G). Our data therefore identify a significant contribution for a single Notch binding site in *Tcf7* initiation during T cell development, but not ILC development.

We further wished to examine the contribution of other candidate factors for the regulation of *Tcf7* expression through the 1 kb region 3. Because of the lack of early T cell precursors in mice deficient for GATA-3 (34), and RUNX (28), we were unable to examine whether these factors contribute to *Tcf7* expression in T cells. Additionally, RUNX is required for the development of ALP, that are upstream of ILC precursors; thus TCF-1 expression during early ILC development cannot be assessed in RUNX deficient mice (30). On the other hand, some EILP are still present in GATA-3 deficient mice (30), which enable us to directly assess TCF-1 expression in these cells. This analysis showed that TCF-1 expression was greatly decreased in GATA-3 deficient TOX^+^ EILP (defined using TOX instead of TCF-1 as before) compared to littermate controls (Figure 7H).

Our data therefore identify a significant contribution for a single Notch binding site in *Tcf7* initiation during T cells development, but not ILC development. Furthermore, GATA-3 significantly contributes to levels of *Tcf7* during initiation of expression in early ILC development.

## Discussion

In this study, we investigated which regulatory elements upstream of *Tcf7* are required for *Tcf7* expression in hematopoietic lineages. We identified a 1 kb region (region 3) located upstream of the *Tcf7* promoter, which contains regulatory elements required for *Tcf7* initiation in T cell and ILC lineages, but not in migratory cDC. We further identified a significant contribution for a conserved Notch binding site located in this region for initiating *Tcf7* expression during early T cell development, but not ILC development.

The 1 kb regulatory element we identified to be required for *Tcf7* initiation in T cells and ILCs was not part of the super-enhancer previously identified for *Tcf7* (25). However, this region contained a high density of transcription factors binding sites, and could correspond to a region bound by a cluster of transcription factors that cooperate to regulate gene expression (35, 36). In particular, this region was bound by many transcription factors required for normal T cell and/or ILC development, such as GATA-3, RUNX, TCF-1, and Notch, which are suggested to be involved in *Tcf7* expression. Notch signaling was thought to be a major controller of *Tcf7* initiation during T cell development (1, 8, 27). By mutating a single Notch binding site, we provide the first evidence that Notch directly contributes to *Tcf7* initiation in T cells. However, contribution of this binding was unexpectedly minor, thus other elements are required to control *Tcf7* initiation in T cells. Notch signaling might further control *Tcf7* initiation in T cells through binding to additional sites, or by inducing expression of other transcription factors such as GATA-3 (29). Because multiple GATA-3 putative binding sites are present in the 1kb *Tcf7* enhancer, examining the role for GATA-3 in directly regulating *Tcf7* expression is challenging. Furthermore, the absence of early T cell precursors in GATA-3 deficient mice (34) prevents the analysis of *Tcf7* expression in ETP in such mice. Interestingly, we found that TCF-1 failed to be properly expressed in GATA-3 deficient early ILC precursors, which are transcriptionally similar to T cell precursors (30). This result may suggest a role for GATA-3 in *Tcf7* initiation or amplification of expression in T cells (29). Consistent with previous work indicating that Notch is not required for early ILC development (37), we did not find a contribution for the Notch binding site in TCF-1 expression in ILC precursors. Future work will aim to identify additional transcriptional controllers involved in *Tcf7* initiation during early T and ILC development, and to understand how they collaborate in this process.

This work additionally identified two regulatory regions (region 1 and region 4) that did not significantly contribute to *Tcf7* initiation in T cells and ILCs, but regulated *Tcf7* expression at later stages of ILC development. This result indicated that distinct regulatory regions may cooperatively regulate *Tcf7* expression at specific developmental stages. The dramatic defect in early T cell and ILC development seen in mice lacking the 1 kb region 3 precluded conclusions on the role of this region beyond *Tcf7* initiation. Indeed, although *Tcf7* was upregulated after the initial defect in expression in both T cells and ILCs, this upregulation could conceivably be the result of compensatory mechanisms that are secondary to the defect in *Tcf7* initiation, and that might not be present in normal (unmutated) cells. Assessing the role of this region after *Tcf7* initiation would thus require the generation of new mouse models allowing conditional deletion after ETP and EILP stages. Importantly, the region 1 we deleted included a previously identified enhancer candidate (19). Although this enhancer was predicted to control *Tcf7* expression in naïve T cells (19), our study did not find support for such a function. Hence our data highlight the difficulty of enhancer prediction, and the necessity to experimentally test enhancer candidates.

By examining TCF-1 expression in various hematopoietic lineages, we found that migratory cDCs expressed high levels of TCF-1. We have not found a role for TCF-1 in the development of cDCs, but it could be involved in cDC function. Interestingly, the regulatory regions required for TCF-1 expression in T cells and ILCs were not important for expression in cDCs. This result indicates the existence of additional regulatory regions, outside the 20 kb region we deleted, that could be required for TCF-1 expression in DCs. Consistently, in cDCs, regions of open chromatin found in T cells and ILCs were absent from the 20 kb region we deleted, but present in the *Tcf7* super-enhancer (25). TCF-1 has been involved in bone formation (38), thermogenesis by brown adipocytes (39), pancreatic β-cell survival and glucose tolerance (40), although the cells in which TCF-1 is expressed are not well defined. Finally, aberrant expression and function of TCF-1 is associated with various diseases, and many cancers (41–45). Understanding the mechanisms by which TCF1 is regulated in these various cell type could enable us to target TCF-1 expression in specific cell types. Such knowledge could be useful to target malignant cells while leaving essential functions intact.

The identification of a shared regulatory element controlling *Tcf7* initiation specifically in T cells and ILC adds to the striking developmental and functional similarities noted between the two lineages (2, 30, 46). Our results additionally indicate that the same regulatory element is used by distinct transcriptional controllers to initiate *Tcf7* expression in T cells and ILCs.

## Data Availability Statement

All datasets generated for this study are included in the article/Supplementary Material.

## Ethics Statement

The animal study was reviewed and approved by NIH Animal Care and Use Committees.

## Author Contributions

CH and AB conceived and directed the research. CH, DK, YD, and YZ performed the experiments. CH and DK analyzed data and made the figures. CH, DK, YW, and PA designed and generated new mouse models. CH, DK, and AB wrote the paper. All authors helped design research, and read and commented on the manuscript.

### Conflict of Interest

The authors declare that the research was conducted in the absence of any commercial or financial relationships that could be construed as a potential conflict of interest. The reviewer GG and handling Editor declared their shared affiliation at the time of review.
