# Supplementary material for: A Shared Regulatory Element Controls the Initiation of Tcf7 Expression During Early T Cell and Innate Lymphoid Cell Developments
Source: Front Immunol. 2020 Mar 20;11:470. doi: 10.3389/fimmu.2020.00470 (PMC7099406; doi:10.3389/fimmu.2020.00470)
Supplement: Supplementary file 1 [file Data_Sheet_1.docx]

Supplementary Material

**Supplementary Figure 1. Design schematic for CRISPR/cas9 guides for mice generation.** (A) ATAC-seq profiles of the region surrounding the *Tcf7* locus in CLP, ETP, DN3, CD4 T cells, B cells, cDC1 and cDC2; ChIP-seq profiles for Notch in a T cell line, TCF-1 in thymocytes, RUNX in thymocytes, GATA-3 in DP, and H3K27ac in naïve CD4 T cells. The blue-boxed region represent the region 1-2 targeted for deletion with CRISPR/cas9 guides, the red-boxed region represent the *Tcf7* superenhancer, the black-boxed region shows a candidate enhancer specific for naïve T cells.

**Supplementary Figure 2. ILCP and ILC2P gating strategies.** Representative gating strategy used to identify (A) ILCP (Lin^BM-^Kit^+^α4β7^+^2B4^+^Flt3^-^Thy1.2^+^IL-7Rα^+^) and (B) ILC2P (Lin^BM-^Kit^-^2B4^lo^Thy1.2^hi^IL-7Rα^+^) by flow cytometry.

**Supplementary Figure 3.** **Analysis of T cells development and TCF-1 expression during early T cells development in enhancer deletion mice.** (A) Flow cytometric analysis of *Tcf7^Δ1/Δ1^*, *Tcf7^Δ2/Δ2^*, *Tcf7^-/-^*, and WT thymocytes. Representative flow plot are shown for total thymocytes (top) and Lin^T-^ thymocytes (bottom). (B) Flow cytometric analysis of *Tcf7^Δ4/Δ4^*, *Tcf7^-/-^*, and WT thymocytes. Representative flow plot are shown for total thymocytes (top) and Lin^T-^ thymocytes (bottom). (C) Quantification of total thymocytes from 7 *Tcf7^Δ1/Δ^* mice, 5 *Tcf7^Δ2/Δ2^* mice, 9 *Tcf7^Δ4/Δ4^* mice, 5 *Tcf7^-/-^* mice, and 6 WT mice pooled from 3 independent experiments. Data are presented as mean +/- SEM. A two-tailed Student’s t-test was used to determine significance. *** *p* < 0.0005. (D) Flow cytometric analysis of TCF-1 expression in T cells from LN of *Tcf7^Δ1/Δ1^*, *Tcf7^-/-^*, and WT mice.

**Supplementary Figure 4.** **Analysis of early ILC development and TCF-1 expression during early ILC development in enhancer deletion mice.** Flow cytometric analysis of *Tcf7^Δ1/Δ1^*, *Tcf7^Δ2/Δ2^*, *Tcf7^Δ4/Δ4^*, *Tcf7^-/-^*, and WT BM cells by intracellular staining. (A) Representative gating strategy used to identify TCF-1^+^ EILP, showing Lin^BM-^Kit^+^α4β7^+^2B4^+^ cells (left). Quantification of frequencies of TCF-1^+^ EILP (right). (B) Quantification of frequencies of ILCP and (C) ILC2P. (A-C) Data are presented as average +/- SEM for n=3 mice of each genotype analyzed in one experiment. A two-tailed Student’s t-test was used to determine significance. * *p* < 0.05, ** *p* < 0.01, *** *p* < 0.005. Data are representative three independent experiments. (D-F) TCF-1 protein expression in (D) TOX^+^ EILP, (E) ILCP and (F) ILC2P. Representative flow plots are shown (left). Data are presented as average of TCF-1 gmfi +/- SEM for n=3 mice of each genotype analyzed in one experiment (right). A two-tailed Student’s t-test was used to determine significance. * *p* < 0.05, ** *p* < 0.01, *** *p* < 0.05. Data are representative of three independent experiments.

**Supplementary Figure 5.** **Involvement of a Notch binding site in TCF-1 initiation during early T cell development.**  (A) Flow cytometric analysis of WT and *Tcf7^NBS/NBS^* thymocytes. Representative flow plot are shown for total thymocytes (left). Quantification of thymocytes numbers is presented as average +/- SEM, for 5 WT mice and 6 *Tcf7^NBS/NBS^* mice pooled from 3 independent experiments (right). A two-tailed Student’s t-test was used to determine significance. (B) TCF-1 intracellular staining on thymocytes from *Tcf7^NBS/NBS^*, *Tcf7*^Δ1-2/Δ1-2^, *Tcf7^-/-^*, and WT mice. Data are representative of three independent experiments. (C) Flow cytometric analysis of TCF-1 protein expression comparing intracellular TCRβ^+^ and intracellular TCRβ^-^ DN3 thymocytes from WT and *Lat^-/-^* mice. Data are representative of three independent experiments. (D-F) Flow cytometric analysis of *Tcf7^NBS/NBS^*, *Tcf7^+/-^*, *Tcf7^-/-^*, and WT BM cells by intracellular staining. Quantification of frequencies of TCF-1^+^ EILP (D), ILCP (E), and ILC2P (F). (D-F) Data are presented as average +/- SEM for n=3 mice of each genotype analyzed in one experiment. A two-tailed Student’s t-test was used to determine significance. * *p* < 0.05, *** *p* < 0.005. Data are representative three independent experiments.
